# Reorganized Functional Networks Underlie Working Memory Deficits After Right‐Hemispheric Stroke

**DOI:** 10.1111/ejn.70336

**Published:** 2025-11-24

**Authors:** Emilie Marti, Sélim Yahia Coll, Naz Doganci, Radek Ptak

**Affiliations:** ^1^ Research Group Spatial Attention, Perception and Action Faculty of Medicine, University of Geneva Geneva Switzerland; ^2^ Division of Neurorehabilitation, Department of Clinical Neurosciences Geneva University Hospitals Geneva Switzerland

**Keywords:** frontoparietal network, functional connectivity, resting‐state fMRI, stroke, working memory

## Abstract

Working memory (WM) is a core component of higher‐order cognition, and its impairment is a common consequence of stroke. While traditional lesion‐symptom mapping highlights focal damage, it often overlooks alterations in large‐scale brain network dynamics. This study investigated WM deficits through functional connectivity (FC) analyses of frontoparietal networks in 34 patients with right hemisphere (RH) stroke and 35 healthy controls. Resting‐state fMRI was used to examine region‐of‐interest and whole‐brain seed‐to‐voxel FC in relation to WM performance on verbal and spatial *N*‐back tasks. Compared to controls, stroke patients exhibited disrupted FC‐WM associations, characterized by reduced intrahemispheric FC between anterior and posterior RH regions, which correlated with poorer WM performance. Notably, enhanced interhemispheric FC, particularly between the right middle and inferior frontal gyri and contralateral parietal cortices, was positively associated with WM accuracy, suggesting compensatory engagement of the intact hemisphere. No performance differences were observed between task modalities, supporting the involvement of domain‐general WM mechanisms. These findings highlight the role of early network‐level reorganization in shaping cognitive outcomes post‐stroke. Specifically, WM deficits appear to result not solely from structural damage but from altered FC patterns, where reduced intrahemispheric connectivity may be mitigated by adaptive interhemispheric recruitment.

## Graphical abstract

This study examined resting‐state functional connectivity underlying working memory after right‐hemisphere stroke. We found disrupted frontoparietal networks, with reduced intrahemispheric and increased interhemispheric connectivity linked to working‐memory deficits. Findings highlight early network reorganization and compensatory recruitment of contralateral regions supporting executive updating processes.

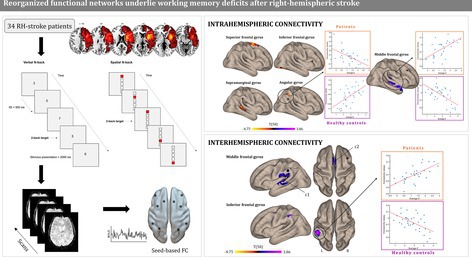

**Abbreviations**
ACA: anterior cerebral artery
AG: angular gyrus
ANOVA: analysis of variance
BOLD: blood oxygenation level dependent
CSF: cerebrospinal fluid
dlPFC: dorsolateral prefrontal cortex
*D*‐prime (*d*′): index of discriminability
(f)MRI: (functional) magnetic resonance imaging
FC: functional connectivity
FDR: false discovery rate methodology
GLM: general linear model
IFG: inferior frontal gyrus
IPL: inferior parietal lobule
LH: left hemisphere
lPFC: lateral prefrontal cortex
MCA: middle cerebral artery
MFG: middle frontal gyrus
MNI: montreal neurological institute
PCA: posterior cerebral artery
PPC: posterior parietal cortex
RH: right‐hemisphere
ROI: region of interest
Rs‐FC: resting state functional connectivity
Rs‐fMRI: resting state functional magnetic resonance imaging
SFG: superior frontal gyrus
SMG: supramarginal gyrus
vlPFC: ventrolateral prefrontal cortex
VOIs: volumetric masks
WM: working memory

## 1 Introduction

Ischemic stroke may lead to significant cognitive impairment and profoundly affect autonomy and quality of life (Hochstenbach et al. 2001; de Haan et al. 2006; Nys et al. 2007; Cumming et al. 2014). Among these cognitive challenges, deficits in working memory (WM) stand out for their frequency, impacting goal‐directed behaviors and overall cognitive functioning (Jaillard et al. 2009). Cognitive models of WM emphasize its role in temporarily maintaining and manipulating relevant information (for a review; Baddeley 1992). Recent models have underscored the significance of attentional control in WM (for a review; Oberauer 2019), but Baddeley and Hitch's 1974 modular model remains one of the most influential. According to this framework, WM is composed of four core components: the central executive, an amodal system that supervises and allocates attention, two modality‐specific storage systems, the phonological loop and the visuospatial sketchpad, and the episodic buffer (Baddeley 2000). A recent meta‐analysis found that stroke survivors experience moderate deficits across all of these subsystems, affecting performance on both low and high‐load WM tasks (Lugtmeijer et al. 2021). These deficits often persist into the chronic recovery phase and are linked to poor functional outcomes, even years after onset (Synhaeve et al. 2015; Lugtmeijer et al. 2021).

There is increasing evidence that focal structural damage can disrupt brain‐wide networks, potentially leading to dysfunction of areas distant from the site of the lesion (Siegel et al. 2016; Adhikari et al. 2017). To investigate network integrity and identify network impairments linked to neurological disorders, resting‐state functional magnetic resonance imaging (rs‐fMRI) has proven to be an invaluable tool (Mirzaei and Adeli 2016; Siegel et al. 2022). One of the most widely used neuroimaging tasks to assess WM is the *N*‐back task (Kirchner 1958), which challenges participants to identify, maintain, manipulate, and update information over time (Miyake and Shah 1999; Miller and Cohen 2001; Marshuetz 2005; Chan et al. 2008; Schmiedek et al. 2009). In the *N*‐back, participants are required to indicate when a currently presented stimulus matches one presented “*N*” trials earlier, with difficulty increasing as “*N*” grows. This task primarily evaluates the updating component of WM, an executive function involving dynamically manipulating and refreshing information in response to new stimuli (Miyake et al. 2000).

The neural correlates of WM have been extensively investigated using task‐based fMRI (Wager and Smith 2003). Meta‐analyses of neuroimaging studies have identified a core network of brain regions engaged during WM tasks, including the dorsolateral (dlPFC) and lateral prefrontal cortex (lPFC), as well as the posterior parietal cortex (PPC) (Owen et al. 2005; Rottschy et al. 2012). Additionally, lesion studies have observed that WM impairments following stroke are associated with damage to these frontoparietal regions (Ptak and Schnider 2004; van Asselen et al. 2006; Baldo and Dronkers 2006; Tsuchida and Fellows 2009; Barbey et al. 2013; Martin et al. 2021). For instance, a recent study from our group investigating modality‐specific effects in WM found that damage to the right lPFC was linked to deficits in verbal WM, while damage to the right temporo‐parietal junction predicted impairments in spatial WM (Marti et al. 2024).

However, functional activation studies struggle to pinpoint brain areas that are both necessary and exclusive to a specific function, while lesion studies demonstrate causal involvement but do not reveal the functional networks underlying that function. To address this gap, we examined brain‐wide functional connectivity (FC) within regions previously linked to WM, and how this connectivity is disrupted following stroke. Using rs‐fMRI, which measures fluctuations in spontaneous neural activity across functionally connected but spatially distant brain regions at rest (Fox and Raichle 2007; Mennes et al. 2011), we investigated whether rs‐FC within distinct brain networks correlates with WM performance on the *N*‐back task, as observed in prior studies of healthy participants (Hampson et al. 2006; Alavash et al. 2015; van Dam et al. 2015). To avoid confounding effects from language impairments, we focused on patients with right hemisphere (RH) damage.

## 2 Methods

### 2.1 Participants

All participants provided informed consent upon enrollment. The study was approved by the Ethics Commission of the Canton of Geneva (Project No. 2018–00135; approval date: May 3, 2018), in accordance with the principles outlined in the Declaration of Helsinki.

The initial sample included 34 patients (mean age = 63.5 ± 12.1 years, 23.5% female) with focal damage to the RH due to a first‐ever brain injury ischemic (*N* = 24) or hemorrhagic stroke (*N* = 10). All patients were examined while hospitalized at the Division of Neurorehabilitation, University Hospitals of Geneva. Eligibility criteria required patients to be able to participate in a standard testing session (in particular, absence of severe visual and language impairment or confusion preventing comprehension of task instructions; ability to provide informed consent; no medical contraindications to perform MRI). Patients with a prior history of vascular, neurological, or psychiatric disorders, as well as those with pure cerebellar or bilateral lesions, were excluded. Due to movement artifacts, the imaging data of five patients exhibited a high proportion of outlier scans (> 10%) in the quality analysis and were therefore excluded from further analysis. Consequently, the final sample consisted of 29 patients, with a mean age of 64.5 ± 11.5 years and 27.6% female participants.

Additionally, a control group of 35 age‐matched healthy individuals participated in the protocol (mean age = 63.5 ± 8.8 years, 48.57% female). Inclusion criteria for the control group included right‐handedness, no history of neurological or psychiatric disorders, and compatibility with MRI procedures. Chi‐square tests of independence showed no significant differences between the patient and control groups in terms of gender distribution [*χ* ^2^(2,64) = 2.934, *p* = 0.087] or age [*χ* ^2^(2,64) = 9.333, *p* = 0.407].

The study consisted of two separate sessions: one for behavioral testing, conducted outside the MRI scanner, and a second for MRI imaging (including both structural and functional scans). For the patient group, behavioral assessments were conducted within 3 months after stroke onset, on average 50.3 ± 30.4 days post‐injury, while structural and functional imaging took place on average 50.3 ± 46.5 days post‐injury. For the control group, the average interval between behavioral testing and the imaging session was 14.7 ± 22.1 days.

### 2.2 Behavioral Methods

#### 2.2.1 Experimental Task

WM performance was assessed using two continuous updating tasks based on the *N*‐back paradigm (Kirchner 1958), where participants identified whether the current item matched the one presented N steps earlier in a sequence of verbal or spatial stimuli. The experimental tasks, developed with E‐Prime 3.0 software (Psychology Software Tools, Pittsburgh, PA), were displayed on a 13.3‐in. HP touchscreen laptop positioned 50 cm away from the participants.

**FIGURE 1:**
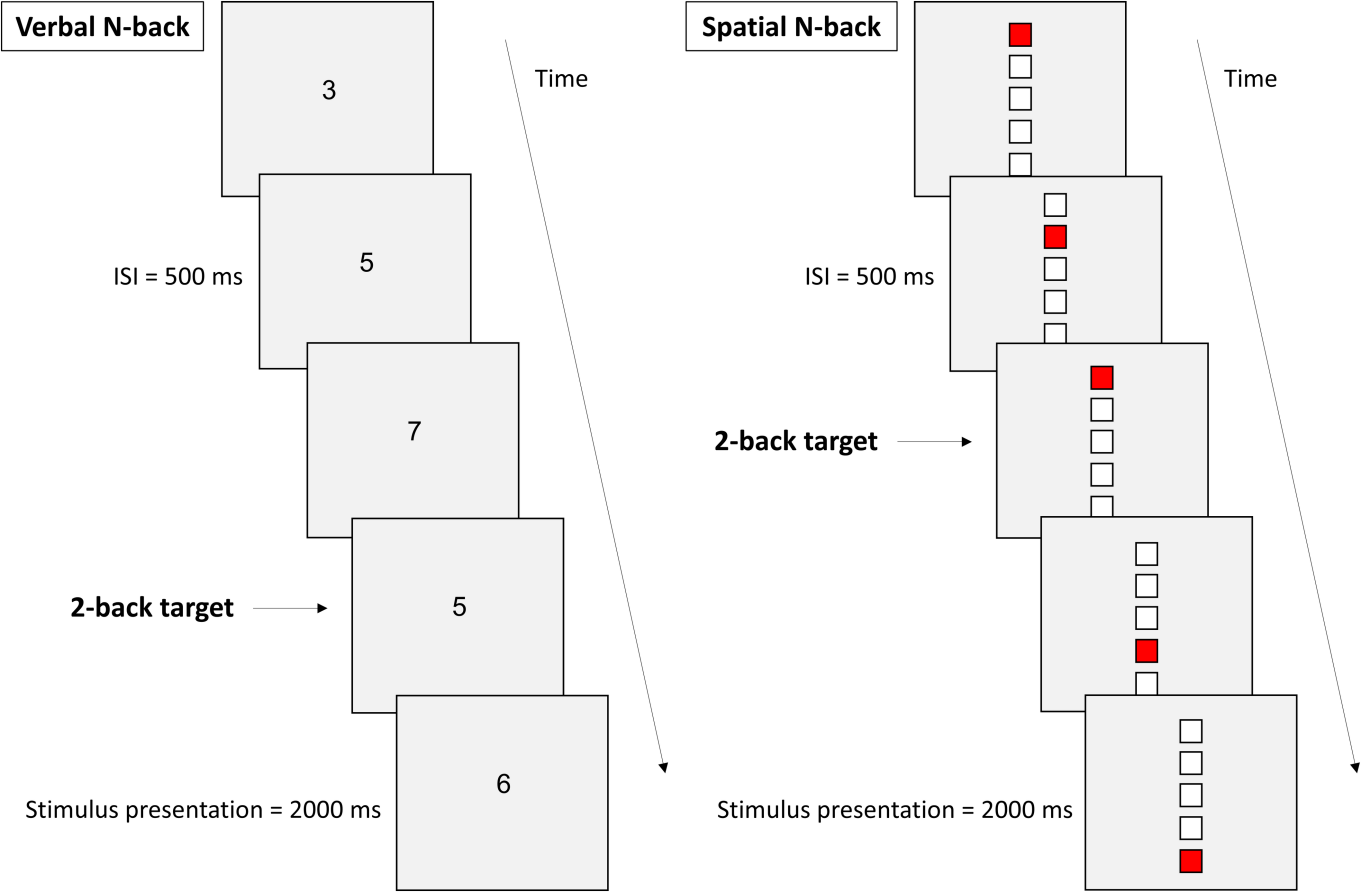
Experimental design and behavioral results. (A) Schematic representation of the sequence and timing of events in the verbal and spatial *N*‐back task (ISI): interstimulus interval.

Participants completed separate adaptations of the *N*‐back task for verbal and spatial information (Figure 1). In the verbal 2‐back, a continuous sequence of digits selected among 3, 4, 5, 6, and 7 appeared in pseudo‐random order, and participants were instructed to press a button on the response box (Cedrus, San Pedro, CA) when the same digit had been presented two positions prior (e.g., *3–5–3*). The spatial 2‐back featured a vertical arrangement of five blank squares, where one square turned red during each trial. Participants had to respond if the red square appeared in the same position as it had two trials earlier (e.g., *second from top—fourth—second*). Although the vertical arrangement of squares could, in principle, encourage verbal encoding strategies (e.g., silently numbering positions), informal feedback indicated that this was rare: only two healthy participants reported using such a strategy, whereas most participants, and all stroke patients, focused on the spatial location of the stimuli.

Accuracy in the *N*‐back tasks was measured using the discriminability index (d‐prime; Haatveit et al. 2010). Responses were categorized as hits (correct responses to targets), false alarms (incorrect responses to distractors), misses (failure to respond to targets), or correct rejections (correct nonresponses to distractors). The main outcome measure of WM performance was the *d*‐prime (*d*′), calculated as *d*′ = Z_hit_—Z_false alarm_, according to Signal Detection Theory. This index reflects the participant's ability to distinguish between targets and distractors, with a higher *d*′‐value indicating superior performance in the WM tasks.

#### 2.2.2 Behavioral Analyses

Behavioral data were analyzed using Statistica software (TIBCO Software Inc.). Mixed analyses of variance (ANOVA) were conducted with *d*′ as the dependent variable. The analysis included a between‐subject factor of Group (Healthy controls, Patients) and within‐subject factors of Task Modality (Verbal, Spatial). Post hoc tests with Bonferroni corrections were performed to examine significant effects.

### 2.3 Neuroimaging Methods

#### 2.3.1 MRI Acquisition

Structural and functional magnetic resonance imaging (MRI) was conducted on a Siemens 3 T Prisma fit scanner with a 64‐channel array coil (Siemens Medical Solutions, Erlangen, Germany). Neuroimaging was performed at the Center for Biomedical Imaging of the University Hospitals of Geneva and Lausanne.

A high‐resolution T1‐weighted MPRAGE sequence (TR: 2300 ms; TE: 1.96 ms; 176 slices; flip angle: 9°; voxel size: 1.0 mm isotropic) was acquired for all participants to enable structural and functional normalization. Additionally, for patients, a T2‐weighted structural scan was collected for lesion delineation (TR: 2400 ms; TE: 226 ms; 176 slices; flip angle: 9°; voxel size: 1.0 mm isotropic). Rs‐fMRI was acquired using a fast echo‐planar imaging sequence (TR: 2000 ms; TE: 35 ms; 320 volumes; flip angle: 85°; voxel size: 5 mm isotropic).

To minimize movement‐related artifacts during acquisition, participants' heads were stabilized with cushions. During the rs‐fMRI scan, participants were instructed to lie still in a dark environment, with their eyes open, fixating on a white cross centered on a black background displayed on an MRI‐compatible screen.

#### 2.3.2 Lesion Delineation and Spatial Normalization

Structural images of patients (T1 and T2) were initially reoriented to the anterior–posterior commissure using Statistical Parametric Mapping (SPM12; Penny et al. 2011). Volumetric masks (VOIs) of each patient's brain lesion were manually delineated on T2‐weighted images using MRIcron software (Rorden et al. 2007) and a digital tablet. These VOIs, along with the T2 scans, were realigned and co‐registered to the corresponding T1‐weighted images before normalization to the standard 1 × 1 × 1 mm age‐specific MNI space (SPM12; Clinical Toolbox; Rorden et al. 2012). To minimize artifacts during normalization of lesioned brains we applied enantiomorphic masking (Nachev et al. 2008). This method replaces damaged tissue with information derived from the homologous area of the intact hemisphere and produces more accurate normalization results than cost function masking, particularly with large cortical lesions. All normalized scans and lesion masks were visually inspected for accuracy, manually corrected where necessary, and smoothed with an 8 mm Gaussian kernel.

**FIGURE 2:**
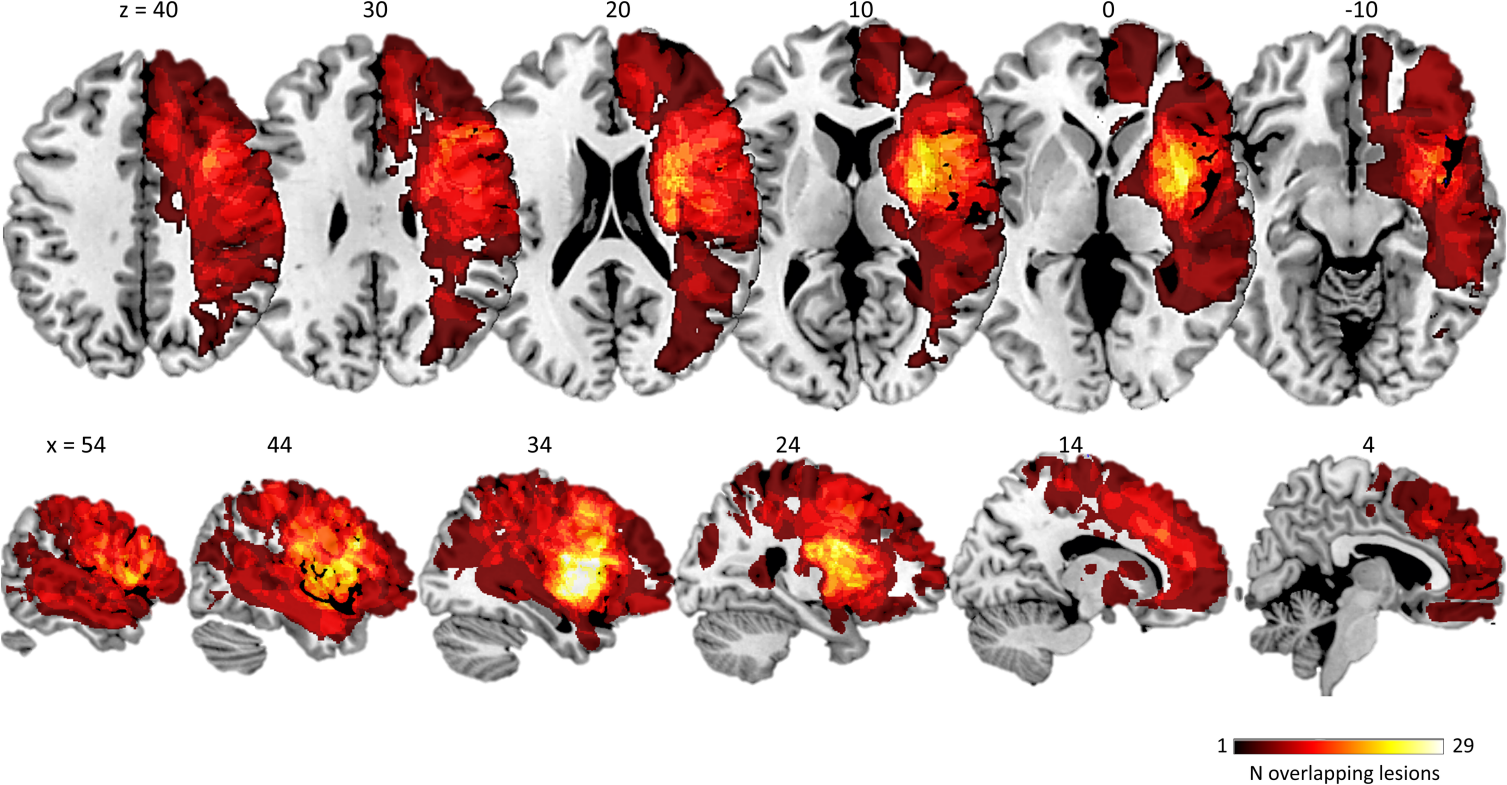
Lesion overlap. Color rendering of brain lesions across all patients. The color scale represents the number of patients with lesioned tissue at each specific voxel. MNI z‐coordinates (for axial slices) and x‐coordinates (for sagittal slices) are indicated above each corresponding section.

To assess lesion distribution, we generated an overlap map of the final patient group (Figure 2). The average lesion volume per patient was 4.51 cm^3^ (SD = 10.64 cm^3^) and the five most frequently affected brain regions were the frontal pole, precentral gyrus, postcentral gyrus, superior frontal gyrus, and middle frontal gyrus. The proportion of patients with lesions affecting each region of interest (ROI) used in the connectivity analysis is as follows: 41% had lesions involving the right superior frontal gyrus (rSFG), 55% the right middle frontal gyrus (rMFG), 45% the right inferior frontal gyrus pars triangularis (rIFGtri), 55% the right inferior frontal gyrus pars opercularis (rIFGop), 34% the right supramarginal gyrus anterior division (rSMGa), 38% the right supramarginal gyrus posterior division (rSMGp), and 24% the right angular gyrus (rAG). Across the 29 patients included, the middle cerebral artery (MCA) was affected in 76% of cases, the anterior cerebral artery (ACA) in 24%, and the posterior cerebral artery (PCA) in 21%. Considering all arterial involvements (*n* = 35 total, as some lesions extended across multiple vascular territories), MCA lesions represented 63%, ACA 20%, and PCA 17%.

#### 2.3.3 Resting‐State fMRI Data Processing

Anatomical and functional data of all participants were processed using a flexible preprocessing pipeline (Nieto‐Castanon 2020) in the CONN toolbox (Whitfield‐Gabrieli and Nieto‐Castanon 2012), which included realignment with correction of susceptibility distortion interactions, slice timing correction, outlier detection, direct segmentation, MNI‐space normalization, and smoothing.

Functional data were realigned and coregistered (Andersson et al. 2001) with the first scan using a 6‐parameter (rigid body) transformation (Friston et al. 1995) and resampled using b‐spline interpolation to correct for motion and magnetic susceptibility artifacts. Temporal misalignment between different slices of the functional data was corrected with the SPM slice‐timing correction procedure (Henson et al. 1999; Sladky et al. 2011).

Outlier scans were identified using the Artifact Detection Tools (Power et al. 2014) when framewise displacement exceeded 0.9 mm or global BOLD signal changes were above five standard deviations, and a reference BOLD image was computed for each subject by averaging all scans excluding outliers. Five patients had more than 10% outlier scans and were excluded from further analysis. The remaining patients had a mean frame displacement of 0.229 ± 0.095 mm, which did not statistically differ from controls (0.196 ± 0.06 mm; *t*(67) = 1.71, *p* = 0.092).

Functional images were normalized into standard MNI space using the transformation matrix derived from the structural normalization, segmented according to tissue class, and resampled to 2 mm isotropic voxels (Calhoun et al. 2017). This process was conducted with SPM's unified segmentation and normalization algorithm (Ashburner and Friston 2005; Ashburner 2007). Importantly, to exclude damaged voxels, functional images were additionally segmented using individualized brain tissue masks created by adding an additional tissue type (“lesion”) to the standard brain masks used by SPM. Therefore, FC was computed exclusively from the actual BOLD signal of nondamaged grey matter, and voxels with possibly corrupt signal were entirely excluded from the analysis (Siegel et al. 2017). Functional images were smoothed using spatial convolution with a Gaussian kernel of 8 mm full width half maximum (FWHM).

Functional data were denoised using a standard denoising pipeline including the regression of potential confounding effects characterized by white matter timeseries (5 CompCor noise components), CSF timeseries (5 CompCor noise components), motion parameters and their first order derivatives (12 factors) (Friston et al. 1996), outlier scans (below 74 factors) (Power et al. 2014), session effects and their first order derivatives (2 factors), and linear trends (2 factors) within each functional run. CompCor (Behzadi et al. 2007; Chai et al. 2012) noise components within white matter and CSF were estimated by computing the average BOLD signal as well as the largest principal components orthogonal to the BOLD average, motion parameters, and outlier scans within each subject's eroded segmentation masks. The BOLD timeseries were filtered with bandpass frequency between 0.008 and 0.09 Hz (Hallquist et al. 2013).

#### 2.3.4 FC Analyses

We performed ROI‐to‐ROI and seed‐to‐voxel analyses to examine FC associated with WM performance. ROI‐to‐ROI analyses examine FC between a subset of brain regions (regarded as network nodes), while seed‐to‐voxel analyses measure FC between a specific ROI and every other voxel of the brain. The two types of analyses are complementary, and coherent findings between the two approaches suggest a particularly robust result.

The analyses focused on five right‐hemispheric frontoparietal regions within the lPFC and inferior parietal lobule (IPL). The selection of these ROIs was guided by a broad body of research on WM updating (Owen et al. 2005; Rottschy et al. 2012). While we also considered lesion findings from our prior work (Marti et al. 2024), the ROIs were independently defined using the Harvard–Oxford Cortical and Subcortical Atlas (Frazier et al. 2005; Desikan et al. 2006; Makris et al. 2006; Goldstein et al. 2007), based on their well‐established role in WM processing.

The selected ROIs included the right superior frontal gyrus (rSFG), right middle frontal gyrus (rMFG), right inferior frontal gyrus (rIFG), right supramarginal gyrus (rSMG) and right angular gyrus (rAG). For ROI‐to‐ROI analyses we also included the five homologous ROIs in the left hemisphere (LH). In addition, to test the specificity of our findings we also performed seed‐to‐voxel connectivity analyses using three alternative seed ROIs for which no FC‐WM relationship was hypothesized: the frontal pole, precuneus, and lateral occipital cortex. Although our primary focus was on RH‐cortical ROIs, seed‐to‐voxel FC was computed across the entire brain. This approach ensured that any potential involvement of LH or subcortical structures, such as the thalamus, could be identified in our analyses.

Group‐level analyses were performed using a General Linear Model (GLM; Nieto‐Castanon 2020). For each individual voxel a separate GLM was estimated, with first‐level connectivity measures at this voxel as dependent variables, and groups as independent variables. Voxel‐level hypotheses were evaluated using multivariate parametric statistics with random effects across subjects and sample covariance estimation across multiple measurements. Inferences were performed at the level of individual clusters (groups of contiguous voxels). Cluster‐level inferences were based on parametric statistics from Gaussian Random Field theory (Worsley et al. 1996; Nieto‐Castanon 2020; Nieto‐Castanon and Whitfield‐Gabrieli 2021). The results were thresholded with a combination of a voxel‐level cluster‐forming threshold of *p* < 0.005 and a cluster‐size threshold corrected for familywise error using *p*‐FDR < 0.05 (Chumbley et al. 2010).

## 3 Results

### 3.1 Behavioral Data

Behavioral results of the two groups in both *N*‐back tasks are shown in Figure 4D. A repeated‐measures ANOVA on 2‐back performance comparing the two groups (Healthy controls, Patients) across two task modalities (Verbal, Spatial) revealed a significant main effect of Group [*F*(1, 62) = 48.8, *p* < 0.001, partial *η* ^2^ = 0.441]. Neither the main effect of Task Modality [*F*(1, 62) = 2.4, *p* = 0.126, partial *η* ^2^ = 0.037], nor the interaction between Group and Modality [*F*(1, 62) = 0.68, *p* = 0.794, partial *η* ^2^ = 0.001] reached significance. Thus, patients were significantly impaired relative to healthy controls irrespective of task modality, and in both groups, performance was not affected by task modality.

In both groups, verbal 2‐back performance correlated significantly with spatial 2‐back performance (controls: *r* = 0.58, *p* < 0.001; patients: *r* = 0.47, *p* = 0.01). To assess whether WM performance was selectively impaired or reflected broader cognitive impairment following stroke we further computed correlations between *N*‐back performance and standard neuropsychological tests (Table 1). *N*‐back performance was nonsignificantly, and mostly negatively correlated with other test results, including forward and backward verbal/spatial memory spans. Notably, the only significant correlations emerged between *N*‐back performance and Matrix Reasoning, a measure of abstract reasoning. This suggests that the observed *N*‐back impairments are specific to WM‐related cognitive processes rather than indicative of a global cognitive impairment post‐stroke.

**TABLE 1:** Pearson correlations between 2‐back tasks and neuropsychological tests.

(*R* value)	Verbal 2‐back	Spatial 2‐back
Forward digit span (Wechsler 2008)	−0.11	−0.17
Backward digit span (Wechsler 2008)	−0.37	−0.22
Forward spatial span (Wechsler 1997)	−0.38	−0.23
Backward spatial span (Wechsler 1997)	−0.26	−0.02
Similarities (Wechsler 2008)	−0.24	−0.19
Bell test left omissions (Gauthier et al. 1989)	−0.17	0.01
Bell test right omissions (Gauthier et al. 1989)	−0.12	0.05
Phonemic fluency; letter P (Godefroy et al. 2010, 2014)	−0.14	−0.14
Semantic fluency; animal naming (Godefroy et al. 2010, 2014)	−0.14	−0.17
5‐point test (Balzer et al. 2011)	−0.27	−0.12
Raven Matrices IQ (Raven and Raven 2003)	0.33	0.32
Matrix reasoning (Wechsler 2008)	0.64 ***	0.66 ***

*Note:* *p*‐values were adjusted using the false discovery rate methodology (FDR); (Benjamini and Yekutieli 2005).

*** Denotes statistical significance at *p* < 0.001.

### 3.2 FC Data

#### 3.2.1 Global Connectivity Patterns

**FIGURE 3:**
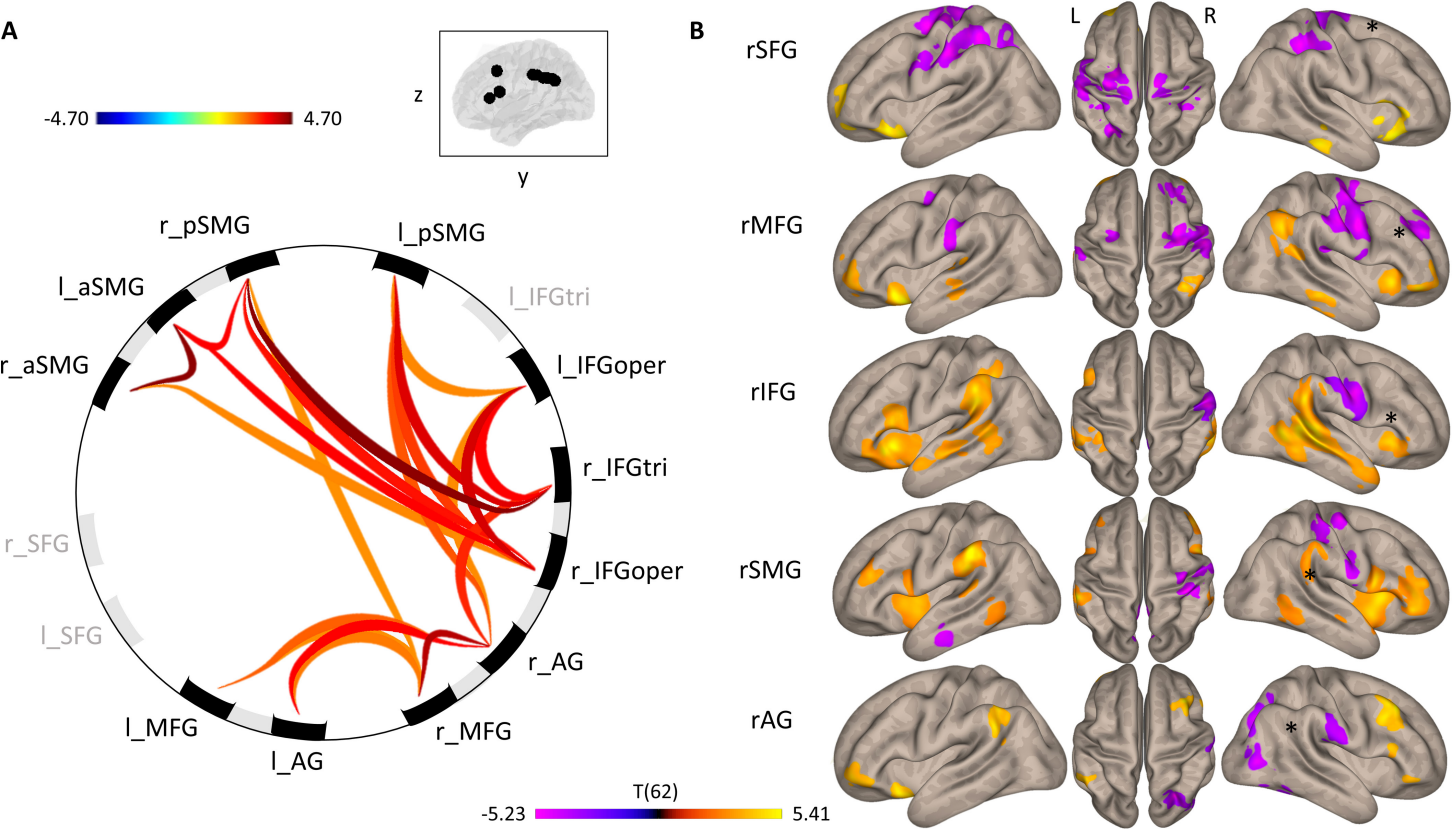
Resting‐state functional connectivity patterns contrasting healthy controls vs. patients. (A) ROI‐to‐ROI connectivity map displaying ROIs with significantly higher correlations in controls than patients (AG: angular gyrus; IFGoper: inferior frontal gyrus, opercular part; IFGtri: inferior frontal gyrus, triangular part; MFG: middle frontal gyrus; SFG: superior frontal gyrus; SMG: supramarginal gyrus). (B) Seed‐to‐voxel analysis focused on RH seed regions. Yellow tones indicate areas where healthy controls exhibited higher connectivity, while purple tones highlight regions where patients demonstrated higher connectivity.

Before investigating specific FC predictors of behavioral performance, we compared ROI‐to‐ROI (Figure 3A) and seed‐to‐voxel connectivity patterns (Figure 3B) between both groups. These analyses showed that stroke fundamentally affected intrahemispheric and interhemispheric brain connectivity. Healthy controls exhibited overall stronger rs‐connectivity between all cortical ROIs except for the left and right SFG and the triangular part of the left IFG (Figure 3A). Whole‐brain seed‐to‐voxel analysis revealed that, compared to patients, healthy controls generally exhibited greater intrahemispheric connectivity with RH regions distant from the seed, and greater interhemispheric connectivity with LH regions that were homotopic to the seed (Figure 3B). In contrast, patients showed higher connectivity with midline ipsilateral or contralateral brain regions involving primary motor, premotor and somatosensory cortices. These findings do not support a general breakdown of FC across the cortex after stroke, but rather an early shift in the connectivity pattern towards decreased FC in associative cortex together with an increase of FC with sensorimotor cortex.

#### 3.2.2 FC as a Predictor of Global WM Performance

Regression analyses were conducted to identify brain regions where FC between identified areas exhibited a significantly higher correlation with global WM performance in healthy controls compared to patients. In our first set of analyses, we were interested in a domain‐general factor reflecting a global WM updating capacity. We therefore sought significant correlations between FC and the *d*′‐value averaged across the two WM tasks.

ROI‐to‐ROI analyses did not yield any significant results, which possibly reflects their focus on predefined, atlas‐based regions. These analyses are inherently more constrained than seed‐to‐voxel analyses, as they only assess connectivity between selected ROIs rather than allowing for whole‐brain exploratory analyses.

##### 3.2.2.1 Intrahemispheric Seed‐Based Connectivity as a Predictor of WM

**FIGURE 4:**
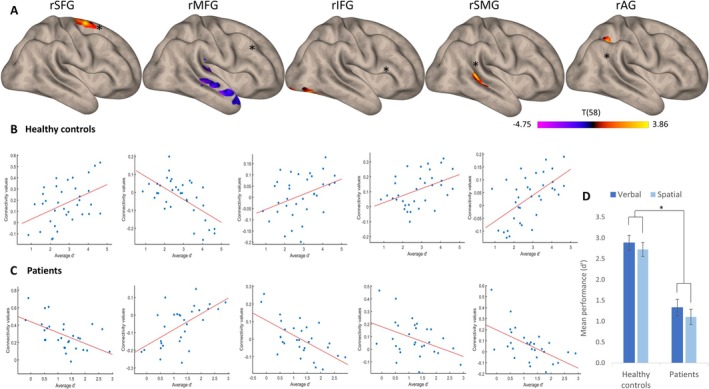
Intrahemispheric seed‐to‐voxel FC patterns predicting WM performance. (A) Connectivity patterns for RH seeds of interest. Yellow tones indicate areas where healthy controls exhibited higher connectivity, while purple tones highlight regions where patients demonstrated higher connectivity. (B) Correlations between FC values and average *d*′ for each seed in healthy controls. (C). Correlations between FC values and average *d*′ for each seed in patients. (D) Mean WM performance (average *d*′) across groups and modalities. Error bars represent 95% confidence intervals.

Seed‐to‐voxel analyses revealed significant differences in FC between groups, with a tendency of healthy controls to exhibit stronger intrahemispheric connectivity (Figure 4A). Most of the significant FC clusters were located close to the seed. Thus, the rSFG seed identified a cluster located within the caudal rSFG (199 voxels; MNI coordinates: +24, −02, +70; FDR‐corrected *p* < 0.020), the rSMG seed a cluster covering the posterior rSMG (300 voxels; MNI coordinates: +42, −38, +10; FDR‐corrected *p* < 0.001) and the rAG seed a cluster in the right superior parietal lobule (169 voxels; MNI coordinates: +24, −42, +36; FDR‐corrected *p* < 0.030). Only the rIFG seed connected with a distant cluster, which was located in the right fusiform gyrus and lateral occipital cortex (279 voxels; MNI coordinates: +44, −66, −18; FDR‐corrected *p* < 0.001).

The only pattern demonstrating a stronger association in patients compared to healthy controls is the FC‐WM association identified through the rMFG seed analysis. This association is observed with the right planum polare (528 voxels; MNI coordinates: +38, 0, −26; FDR‐corrected *p* < 0.001) and the superior temporal cortex.

Correlations within these clusters showed opposing patterns between healthy controls and patients (Figure 4B,C), with a predominantly positive relationship in healthy controls, and a negative relationship in patients (except for the rMFG seed). Pearson correlations for each seed‐cluster pair in both groups are provided in Table 2. A positive correlation indicates that higher seed‐to‐voxel FC is associated with better WM performance. This suggests that intrahemispheric connectivity generally supports WM function in healthy controls while it is inefficient in supporting WM function in patients. The exception to this pattern was the connectivity between rMFG and the right temporal cortex.

**TABLE 2:** Pearson correlations for each seed‐cluster pair in healthy controls and patients, focusing on global WM performance.

Group	RH‐seed of interest	Cluster	Correlation
Healthy controls			
Intrahemispheric	Superior frontal gyrus	Right superior frontal gyrus	0.43
	Middle frontal gyrus	Right planum polare	−0.61
	Inferior frontal gyrus	Right occipital fusiform gyrus	0.37
	Supramarginal gyrus	Right posterior supramarginal gyrus	0.43
	Angular gyrus	Right superior parietal lobule	0.58
Interhemispheric	Middle frontal gyrus	Left planum temporale	−0.56
		Anterior cingulate gyrus	−0.62
	Inferior frontal gyrus	Left anterior supramarginal gyrus	−0.60
Patients			
Intrahemispheric	Superior frontal gyrus	Right superior frontal gyrus	−0.55
	Middle frontal gyrus	Right planum polare	0.58
	Inferior frontal gyrus	Right occipital fusiform gyrus	−0.63
	Supramarginal gyrus	Right posterior supramarginal gyrus	−0.41
	Angular gyrus	Right superior parietal lobule	−0.57
Interhemispheric	Middle frontal gyrus	Left planum temporale	0.67
		Anterior cingulate gyrus	0.46
	Inferior frontal gyrus	Left anterior supramarginal gyrus	0.51

Analysis of FC with three control ROIs (frontal pole, precuneus, lateral occipital cortex) did not yield any significant FC‐WM relationships, confirming the specificity of our target ROIs for WM.

##### 3.2.2.2 Interhemispheric Seed‐Based Connectivity as a Predictor of WM

**FIGURE 5:**
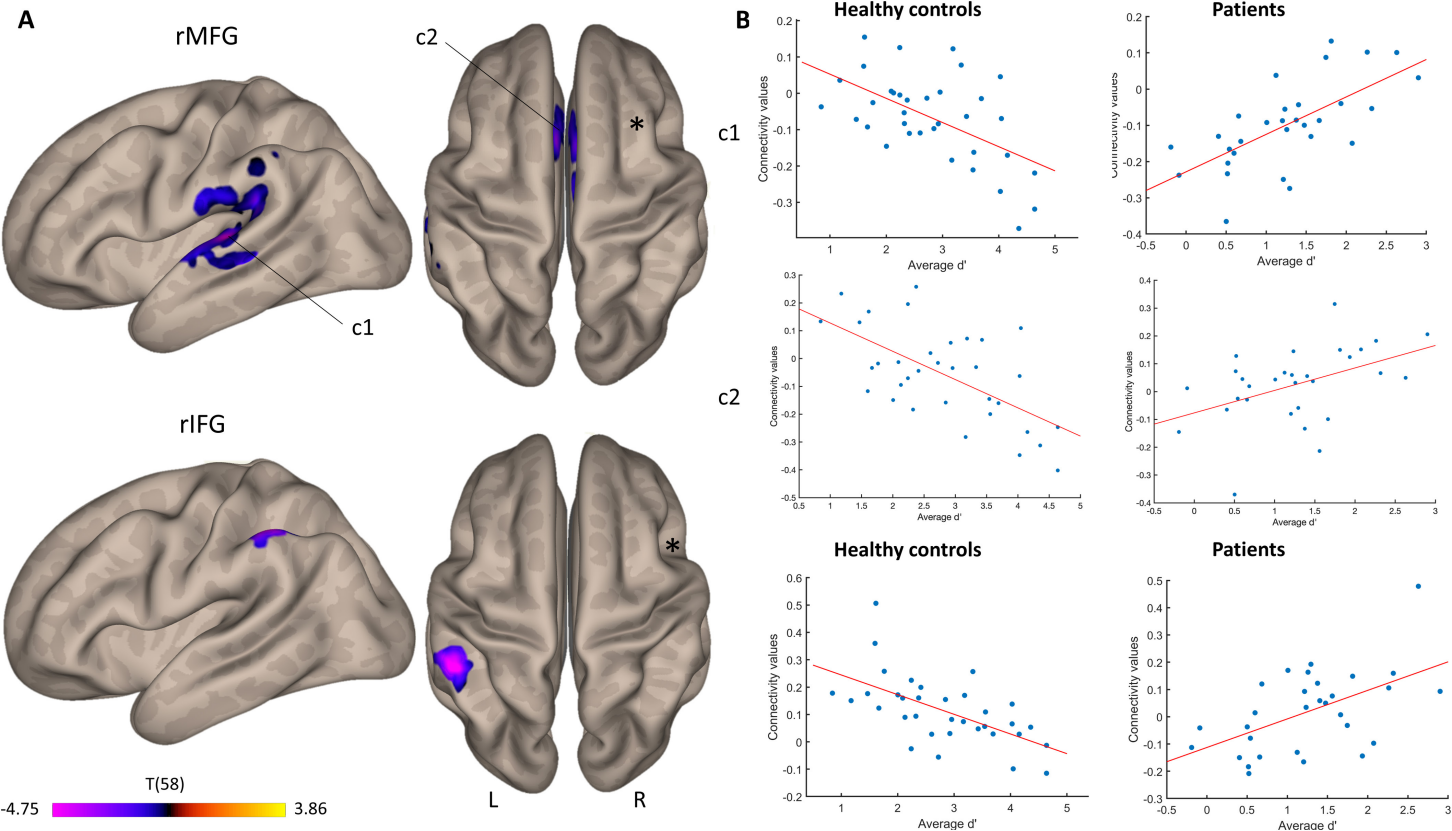
Interhemispheric seed‐to‐voxel FC patterns predicting WM performance. (A) Connectivity patterns for RH seeds of interest. Purple tones highlight regions where patients demonstrated higher connectivity. (B) Correlations between FC values and average *d*′ for the two seeds of interest in healthy controls and patients.

Seed‐to‐voxel analyses revealed significant group differences in FC, with patients showing a stronger relationship between interhemispheric connectivity and behavior than healthy controls (Figure 5A). The rMFG seed identified the left planum temporale (1043 voxels; MNI coordinates: −42, −30, +04; FDR‐corrected *p* < 0.000), the left anterior SMG, and the anterior cingulate gyrus (705 voxels; MNI coordinates: −02, +12, +36; FDR‐corrected *p* < 0.000). FC between the rIFG seed and the left anterior SMG (249 voxels; MNI coordinates: −54, −36, +48; FDR‐corrected *p* < 0.002) also predicted performance better in patients than in controls.

As for intrahemispheric FC, opposing correlation patterns emerged between healthy controls and patients (Figure 5B, Table 2). The FC‐WM correlations were positive in patients and negative in controls, indicating that they were beneficial to performance in the former, but deleterious in the latter.

### 3.3 Verbal and Spatial WM

#### 3.3.1 FC in Verbal and Spatial WM

To assess modality‐specific effects in WM, we performed separate ROI‐to‐ROI and seed‐to‐voxel analyses for verbal and spatial WM tasks. The former analysis yielded no significant results for either modality. In contrast, seed‐to‐voxel analyses revealed patterns consistent with global WM findings, whereby the association between FC and behavior was positive in healthy controls for intrahemispheric connectivity, and positive in patients for interhemispheric connectivity. Table 3 presents the MNI coordinates for each seed‐cluster pair, analyzed separately for verbal and spatial WM. Regions identified in these analyses largely overlapped with those from the global WM results, especially for the rMFG, rIFG, and rSMG seeds. Additionally, FC‐WM correlations showed the same opposing patterns between groups: negative correlations in healthy controls (stronger connectivity linked to poorer WM performance) and positive correlations in patients (stronger connectivity linked to better WM performance).

**TABLE 3:** Results of the seed‐to‐voxel analysis for the contrast healthy controls > patients for verbal and spatial WM separately. Anatomical labels of significant clusters are indicated according to the Harvard‐Oxford Atlas.

Modality	RH‐seed of interest	Cluster	MNI coordinates			Cluster size
			*x*	*y*	*z*	
Verbal	Superior frontal gyrus	Left temporal occipital fusiform cortex	−40	−52	−22	320
	Middle frontal gyrus	Heschl's gyrus	−56	−12	+04	1270
		Left inferior temporal gyrus	−50	−58	−10	409
		Right supplementary motor cortex	+12	−04	+48	293
		Right planum polare	+38	+00	−26	238
		Cerebellum	+18	−72	−52	171
		Anterior cingulate gyrus	+00	+12	+32	170
	Inferior frontal gyrus	Left lateral occipital cortex	−28	−80	+08	906
		Cerebellum	+22	−74	−52	850
		Left anterior supramarginal gyrus	−54	−36	+48	302
		Left superior frontal gyrus	−18	+06	+62	267
		Right frontal pole	+16	+68	+28	218
		Left middle frontal gyrus	−40	+10	+38	184
	Supramarginal gyrus	Left subcallosal cortex	−12	+30	−14	191
		Right frontal pole	+26	+66	+26	183
Spatial	Superior frontal gyrus	Heschl's gyrus	+38	−24	+04	264
		Left superior frontal gyrus	+24	−02	+70	177
	Middle frontal gyrus	Anterior cingulate gyrus	+00	+12	+36	528
		Heschl's gyrus	+40	−26	+04	271
		Left planum temporale	−42	−30	+04	250
		Left temporal pole	−36	+02	−20	171
	Supramarginal gyrus	Right posterior supramarginal gyrus	+40	−38	+12	283

Notably, none of the observed FC changes were linked to time since stroke onset, as intra‐ and interhemispheric FC showed no significant correlations with this variable.

Taken together, these findings highlight the contrast between the groups: healthy controls show robust intrahemispheric connectivity supporting efficient WM performance, while patients exhibit disrupted intrahemispheric connectivity and increased interhemispheric recruitment to support WM function.

## 4 Discussion

Our study demonstrates that deficits in the *N*‐back task, which reflect key WM processes, are linked to alterations in rs‐FC within the first 3 months following a stroke. While network reorganization is an ongoing process that extends beyond this timeframe (Rossini et al. 2003), our findings indicate that FC reorganization relates to WM performance already in the subacute phase.

To minimize confounding effects, we excluded patients with LH stroke to avoid language impairments and controlled for spatial cognition deficits by presenting all stimuli centrally. The results show that, regardless of task modality, RH stroke leads to impaired N‐back performance and distinct alterations of both intra‐ and interhemispheric FC. These findings align with prior research emphasizing the role of network integration in WM (Osaka et al. 2021). For instance, higher FC is linked to better WM outcomes (Hampson et al. 2006), and increased modularity within rs‐networks predicts individual variability in visual WM tasks (Stevens et al. 2012). Additionally, studies have shown that increasing task complexity enhances network integration, suggesting that network architecture adapts dynamically to cognitive demands (Kitzbichler et al. 2011). Together, these observations highlight the importance of FC in understanding cognitive deficits after stroke, as it reflects the efficiency of communication between regions crucial for WM.

### 4.1 Decreased Intrahemispheric Connectivity

Seed‐to‐voxel analyses in healthy controls revealed stronger intrahemispheric connectivity within the right‐lateralized frontoparietal networks, particularly between the lPFC and PPC. FC between these regions positively correlated with improved performance on the *N*‐back task, underscoring the significance of these connections in supporting WM. These findings are consistent with previous meta‐analyses of activation studies, which have identified the prefrontal and parietal regions as core components of WM networks (Owen et al. 2005; Rottschy et al. 2012). Indeed, the lateral prefrontal and parietal cortices are reliably engaged across a wide range of WM tasks (Glabus et al. 2003; Zou et al. 2013; van Dam et al. 2015), including those employing visual WM paradigms (Rama et al. 2001; Todd and Marois 2004).

The dlPFC and ventrolateral (vlPFC) prefrontal cortices, together with the IPL, contribute to key WM processes, such as storage and manipulation of information (Baldo and Dronkers 2006; Champod and Petrides 2007; Corbetta et al. 2000), as well as the updating of mental representations (Braver et al. 1997; Cohen et al. 1997; Baddeley 2003). In contrast to the robust and efficient patterns of connectivity observed in healthy controls, patients exhibited reduced intrahemispheric connectivity. These disruptions, although associated with structural damage identified in our previous connectome‐based study (Marti et al. 2024), also reflect a broader disintegration of FC networks, including nodes located distal to the primary lesion site (Golestani et al. 2014). In both structural and functional domains, disruptions within right frontoparietal pathways, particularly between the dlPFC and IPL, emerge as key predictors of WM deficits, underscoring the central role of this network in WM updating. Importantly, while the structural approach delineated the anatomical substrates of disconnection, the present FC findings capture a wider network‐level dysfunction, including the upregulation of connections to regions remote from the lesion site. These findings highlight the complementary insights provided by the two approaches.

The observed connectivity patterns illuminate distinct mechanisms underlying the efficiency of WM. In healthy controls, significant correlations between FC and WM performance suggest that synchronized activity within RH networks supports WM processes. Notably, the right lPFC and PPC are not only strongly activated during various WM tasks but also exhibit rs‐functional interactions that predict *N*‐back task performance. These findings emphasize the importance of right frontoparietal connectivity for mental monitoring and updating processes (Baddeley 2003; Corbetta et al. 2000; Owen 1997). Supporting this, a recent rs‐fMRI study demonstrated that connectivity between these regions is closely associated with enhanced WM performance (H. Liu, Yu, et al. 2017).

In contrast, patients exhibited an inverse relationship, with greater connectivity within disrupted networks correlating with poorer WM performance. This negative correlation may reflect compensatory but inefficient processing within the lesioned hemisphere (J. Liu, Wang, et al. 2017). An exception was found for the rMFG, the right planum polare and the superior temporal cortex, where a positive FC‐WM association emerged in patients only. This isolated finding may reflect task‐specific recruitment of frontotemporal pathways or compensatory engagement of additional cognitive resources due to dysfunction in primary frontoparietal networks, a phenomenon observed in cognitive and psychiatric disorders (Menon 2011; Hillary et al. 2015).

Overall, these findings suggest that post‐stroke WM performance is influenced by shifts in functional networks, wherein adaptive yet suboptimal patterns of FC arise in response to structural damage. However, given the early poststroke timeframe, it is possible that these patterns represent transient connectivity changes rather than fully stabilized network reorganization. This aligns with findings suggesting that stroke‐induced functional changes are highly dynamic in the subacute phase (Carter et al. 2012; Siegel et al. 2017).

### 4.2 Compensatory Interhemispheric Connectivity

In addition to lesion location, cognitive impairment following stroke is closely linked with disruptions in FC, highlighting the importance of distributed network connectivity for behavioral performance (Siegel et al. 2016; Adhikari et al. 2017; Lugtmeijer et al. 2021). Stroke‐induced changes extend beyond the injured hemisphere, affecting connectivity with contralateral regions and leading to widespread alterations in network dynamics (Alstott et al. 2009; Van Den Heuvel and Pol 2010; Gratton et al. 2012; Hermundstad et al. 2013). Prior studies have demonstrated that stroke disrupts both intrahemispheric FC within the affected hemisphere and interhemispheric FC between homotopic and heterotopic areas (Siegel et al. 2016; Ptak et al. 2020).

Our findings revealed more nuanced changes in FC. As expected, many areas exhibited decreased connectivity within the RH and between hemispheres. However, we also observed increases in both intrahemispheric and interhemispheric connectivity compared to healthy controls, reflecting early compensatory mechanisms. Notably, FC increases during rest were predominantly localized to midline regions, including motor, premotor and somatosensory cortices. Furthermore, enhanced FC between the right MFG, IFG, and contralateral parietal cortex was positively correlated with better WM performance. This early‐stage reorganization, including the recruitment of interhemispheric connections, appears to play a compensatory role in supporting the manipulation and updating of mental information.

Compensatory connectivity within large‐scale networks has been demonstrated to play a pivotal role in supporting various cognitive functions, including language and spatial cognition (Carter et al. 2010; Ptak et al. 2020). The engagement of interhemispheric communication likely enables the brain to adapt to structural damage, facilitating the maintenance of cognitive performance through the utilization of alternative communication pathways.

### 4.3 Domain‐General Network for WM Updating

Prior research has highlighted the critical role of frontoparietal networks, particularly involving the lPFC and IPL, in supporting WM storage and manipulation. Our findings extend these observations by underscoring the involvement of these networks in executive WM processes. The connectivity patterns observed in our study are consistent with meta‐analyses demonstrating the activation of frontoparietal regions in tasks requiring WM updating (Owen et al. 2005; Nee et al. 2013; Satake et al. 2024).

The *N*‐back task engages a wide array of cognitive processes, including sequencing and temporal updating of mental representations, consolidating information, inhibiting distractions, and selecting and organizing responses (Volle et al. 2008). These processes are strongly linked to the lPFC, particularly the mid‐dlPFC PFC, a region critical for managing complex cognitive operations and integrating diverse attentional and executive demands (Owen et al. 1998; Rowe et al. 2000; Pochon et al. 2001; Sakai et al. 2002; Volle et al. 2005; Postle 2006). Furthermore, recent studies have shown that rs‐activity in the MFG and IPL positively correlates with *N*‐back performance in healthy participants, reinforcing the significance of these regions in updating and other central WM functions (Zou et al. 2013).

Consistent with functional imaging studies that report no clear lateralization effects in WM tasks (Nystrom et al. 2000; Owen et al. 2005), our behavioral analyses revealed that both verbal and spatial *N*‐back performance was similarly impaired following RH stroke. This finding aligns with prior research demonstrating that lesions in frontal and parietal regions disrupt verbal and spatial WM to a comparable degree (van Dam et al. 2015; Lugtmeijer et al. 2021). These results suggest that stroke‐related impairments are not modality‐specific but instead reflect disruptions to a domain‐general WM mechanism, as further supported by the significant correlations observed between the two *N*‐back tasks.

This evidence highlights the integrative and adaptable nature of frontoparietal networks in supporting cognition. The domain‐general role of these networks allows them to flexibly engage in various cognitive processes across modalities and tasks, making these processes particularly vulnerable to structural or functional damage. These conclusions are further supported by studies identifying the frontoparietal network as a cornerstone of domain‐general cognitive functions (Zhou et al. 2023).

## 5 Conclusions

Our findings demonstrate that RH stroke leads to bilateral disruptions and adaptations in FC within frontoparietal networks. While network changes likely continue beyond the subacute phase, our results indicate that WM deficits are associated with early reorganization involving both hemispheres. These connectivity shifts predict WM deficits and reflect maladaptive and compensatory processes.

These results underscore the critical role of integrative brain networks in WM, reinforcing that higher‐order cognition relies on network connectivity rather than isolated regions. Furthermore, changes in frontoparietal connectivity predict WM performance and reflect neural compensatory mechanisms in response to brain injury.

Finally, our study highlights that stroke‐related WM impairments affect processes across task modalities, supporting the view that frontoparietal networks are essential for domain‐general cognitive functions. This emphasizes the importance of targeting these networks in therapeutic interventions aimed at mitigating cognitive deficits after stroke.

## Author Contributions

**Emilie Marti:** data curation, formal analysis, investigation, methodology, project administration, visualization, writing – original draft. **Sélim Yahia Coll:** data curation, formal analysis, investigation, methodology, project administration. **Naz Doganci:** data curation, formal analysis, investigation, methodology, project administration. **Radek Ptak:** conceptualization, data curation, formal analysis, funding acquisition, investigation, methodology, project administration, resources, supervision, validation, writing – review and editing.

## Funding

Schweizerischer Nationalfonds zur Förderung der Wissenschaftlichen Forschung, 32003B–219206.

## Conflicts of Interest

The authors declare no conflicts of interest.

## Data Availability

The data used in this study areavailable in the Yareta repository at the following link: https://doi.org/10.26037/yareta:lppbbycbtnduljmb5qlpjfg2ti.
